# Spin Hall Effect in Paraxial Vectorial Light Beams with an Infinite Number of Polarization Singularities

**DOI:** 10.3390/mi14071470

**Published:** 2023-07-21

**Authors:** Alexey A. Kovalev, Victor V. Kotlyar, Anton G. Nalimov

**Affiliations:** 1 Image Processing Systems Institute of the RAS—Branch of FSRC “Crystallography & Photonics” of the RAS, 151 Molodogvardeyskaya St., 443001 Samara, Russia; alanko@ipsiras.ru (A.A.K.); kotlyar@ipsiras.ru (V.V.K.); 2 Technical Cybernetics Department, Samara National Research University, 34 Moskovskoe Shosse, 443086 Samara, Russia

**Keywords:** optical spin Hall effect, paraxial light beam, polarization singularity, radial polarization, Gaussian beam, spin angular momentum

## Abstract

Elements of micromachines can be driven by light, including structured light with phase and/or polarization singularities. We investigate here a paraxial vector Gaussian beam with an infinite number of polarization singularities residing evenly on a straight line. The intensity distribution is derived analytically and the polarization singularities are shown to exist only in the initial plane and in the far field. The azimuthal angle of the polarization singularities is shown to increase in the far field by π/2. We obtain the longitudinal component of the spin angular momentum (SAM) density and show that it is independent of the azimuthal angle of the polarization singularities. Upon propagation in free space, an infinite number of C-points is generated, where polarization is circular. We show that the SAM density distribution has a shape of four spots, two with left and two with right elliptic polarization. The distance to the transverse plane with the maximal SAM density decreases with decreasing distance between the polarization singularities in the initial plane. Generating such alternating areas with positive and negative SAM density, despite linear polarization in the initial plane, manifests the optical spin Hall effect. Application areas of the obtained results include designing micromachines with optically driven elements.

## 1. Introduction

Properties of light beams, and, in particular, optical vortices, can differ in near and far fields of diffraction. For instance, in [1], fractional-order optical vortices were studied in the near field. Such vortices contain chains of alternating ± first-order vortices, which disappear in the far field. As was demonstrated in [2], an optical vortex generated by a refractive spiral phase plate has an asymmetric shape in the Fresnel diffraction zone. Another work [3] investigated near-field diffraction of a Gaussian beam by fork gratings with different topological charges, and it was found that the generated optical vortices reside on spiral lines around the ± first diffraction orders. In far-field diffraction, transformation was found of the initially fractional topological charge [4,5]. In addition, in the far field (in the lens focus) of optical vortices, the spin Hall effect arises [6,7], i.e., alternating pairs of areas are generated in the focus with left and right circular polarization, despite linear polarization of the initial beam. This effect was discovered both for homogeneous linear polarization [8] and for inhomogeneous polarization (cylindrical) [9] of the initial field.

A natural generalization of a vortex light beam is a beam with several vortices. A seminal work with multiple vortices [10] investigated Gaussian beams with optical vortices located in the transverse plane arbitrarily. These beams are described by finite products with the number of multipliers equal to the number of vortices. Another work [11] describes propagation-invariant light fields with an arbitrary envelope analytical function in a closed form, whose zeros define positions of vortices in the beam. Based on [10], a light field can be constructed that has phase singularities residing on a circle [12]. In our paper [13], we investigated a similar field, but with polarization singularities on a circle. Recently we found that the spin Hall effect also arises in such fields [14], but in the Fresnel diffraction zone, rather than in the far field.

Besides the finite number of optical vortices, the approach from [11] allows for obtaining a light field with an infinite array of optical vortices, residing on a straight line [15]. Such fields have an infinite topological charge [16], can be generated by non-coaxial superposition of two tilted Gaussian beams [17], and are identified by density of the optical vortices, rather than by the topological charge, which can be measured interferometrically [15].

In this work, we study a vectorial Gaussian beam where, instead of phase singularities, an infinite number of polarization singularities reside on a straight line. The polarization singularities index (Poincaré–Hopf index) [18,19] of such a beam is shown to be also infinite. We found that the azimuthal angle of the polarization singularities [20] increases in the far field by *π*/2, i.e., initial radial polarization is converted to azimuthal and vice versa. It is demonstrated that when such a beam propagates in space, two pairs of areas are generated in the Fresnel zone with nonzero density of the longitudinal component of the spin angular momentum (SAM), despite linear polarization in the initial plane; i.e., the optical spin Hall effect arises.

## 2. Paraxial Light Fields with an Infinite Number of Phase or Polarization Singularities

In [11], the following solution to the paraxial Helmholtz equation has been obtained (Equation (17) in [11]):

(1) $$E\left(r,\varphi ,z\right)=\frac{1}{q}\exp\left(-\frac{r^{2}}{qw_{0}^{2}}\right)f\left(\frac{re^{i\varphi }}{qw_{0}}\right),$$

where (*r*, *φ*, *z*) are the cylindrical coordinates, *w*_0_ is the waist radius of the Gaussian beam, *q* = 1 + *iz*/*z*_0_, and *f*(ξ) is an arbitrary entire analytical function. The field (1) does not change its intensity structure on propagation in space. It is only widened |*q*| = [1 + (*z*/*z*_0_)]^1/2^ times and rotated around the optical axis by an angle arg(*q)* = arctan(*z*/*z*_0_).

This general expression allows for obtaining a solution of the paraxial Helmholtz equation with infinite or a finite number of optical vortices. For instance, if *f*(ξ) = cos(*w*_0_ξ/*α*_0_), then the vortices reside evenly along a straight line [15]:

(2) $$E\left(r,\varphi ,z\right)=\frac{1}{q}\exp\left(-\frac{r^{2}}{qw_{0}^{2}}\right)\cos\left(\frac{re^{i\varphi }}{\alpha_{0}q}\right).$$

This field is an example of light fields with an infinite topological charge [16]. In the initial plane of such a field, optical vortices reside in points with the Cartesian coordinates *x_p_* = *α*_0_(*π*/2 + *πp*), *y* = 0 with *p* being integer numbers.

It has long been known [21] that cylindrical polarization can be represented as a superposition of ± first-order optical vortices with opposite circular polarization. For the Jones vectors, such representation can be written as

(3) $$\left[\begin{array}{c} \cos\left(\varphi +\delta \right)\\ \sin\left(\varphi +\delta \right) \end{array}\right]=\frac{1}{2}\exp\left(i\varphi +i\delta \right)\left(\begin{array}{c} 1\\ -i \end{array}\right)+\frac{1}{2}\exp\left(-i\varphi -i\delta \right)\left(\begin{array}{c} 1\\ i \end{array}\right).$$

where *δ* is the azimuthal angle of cylindrical polarization (if *δ* = 0 or if *δ* = *π*/2, polarization is respectively radial or azimuthal) [20].

Then, if we use the same Jones vectors, but instead of the multipliers *e^iφ^* and *e*^−*iφ*^ we substitute the field (2) with the cosine argument *re^iφ^* and *re*^−*iφ*^ respectively, we construct a vector field with an infinite number of the polarization singularities:

(4) $$\begin{array}{l} E\left(x,y,z\right)=\frac{1}{2q\sqrt{W_{0}}}\exp\left(-\frac{x^{2}+y^{2}}{qw_{0}^{2}}\right)\\ \;\;\times \left[\exp\left(i\delta \right)\cos\left(\frac{x+iy}{\alpha_{0}q}\right)\left(\begin{array}{c} 1\\ -i \end{array}\right)+\exp\left(-i\delta \right)\cos\left(\frac{x-iy}{\alpha_{0}q}\right)\left(\begin{array}{c} 1\\ i \end{array}\right)\right], \end{array}$$

where (*x*, *y*) are the Cartesian coordinates in the transverse plane and *W*_0_ is a multiplier introduced for normalizing the beam energy (i.e., in order to make it equal to the unit). This multiplier can be obtained from an expression for the energy of the scalar field (2) [15]:

(5) $$W_{0}=\frac{\pi w_{0}^{2}}{2}\cosh\left(\frac{w_{0}^{2}}{2\alpha_{0}^{2}}\right).$$

Superposition (4) consists of two beams. Upon propagation in space, one of them is rotated clockwise and the other counterclockwise. Note that such a field (4) does not retain the intensity structure during propagation; that is, it is not structurally stable, because there are optical vortices with different signs in it.

**Figure 1:**
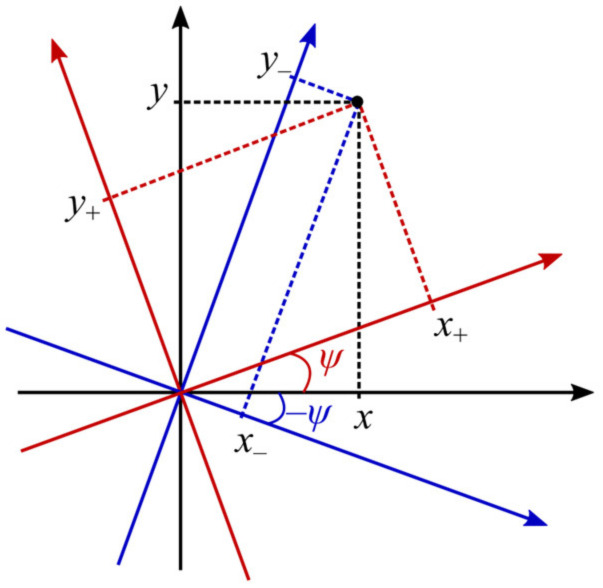
Coordinate systems (*x*_+_, *y*_+_) and (*x*_−_, *y*_−_).

For a compact description of such propagation, we introduce two rotated coordinate systems (Figure 1):

(6) $$\left\{\begin{array}{l} x_{\pm }=\left(x\cos\psi \pm y\sin\psi \right)/\left(\alpha_{0}\left|q\right|\right),\\ y_{\pm }=\left(y\cos\psi \mp x\sin\psi \right)/\left(\alpha_{0}\left|q\right|\right), \end{array}\right.$$

with *ψ* = arctan(*z*/*z*_0_) being the Gouy phase.

Then the complex amplitude (4) can be rewritten as

(7) $$\begin{array}{l} E\left(x,y,z\right)=\frac{1}{2q\sqrt{W_{0}}}\exp\left(-\frac{x^{2}+y^{2}}{qw_{0}^{2}}\right)\\ \;\;\times \left[\exp\left(i\delta \right)\cos\left(x_{+}+iy_{+}\right)\left(\begin{array}{c} 1\\ -i \end{array}\right)+\exp\left(-i\delta \right)\cos\left(x_{-}-iy_{-}\right)\left(\begin{array}{c} 1\\ i \end{array}\right)\right], \end{array}$$

or in a matrix form

(8) $$E\left(x,y,z\right)=\frac{1}{2q\sqrt{W_{0}}}\exp\left(-\frac{x^{2}+y^{2}}{qw_{0}^{2}}\right)\left[\begin{array}{cc} \cos\delta & -\sin\delta \\ \sin\delta & \cos\delta \end{array}\right]\left[\begin{array}{cc} 1 & 1\\ -i & i \end{array}\right]\left[\begin{array}{c} \cos\left(x_{+}+iy_{+}\right)\\ \cos\left(x_{-}-iy_{-}\right) \end{array}\right]$$

where the matrix

(9) $$S=\left[\begin{array}{cc} 1 & 1\\ -i & i \end{array}\right]$$

converts phase singularities (optical vortices) into polarization singularities (radial polarization), while the matrix

(10) $$R=\left[\begin{array}{l} \cos\delta \;-\sin\delta \\ \sin\delta \;\cos\delta \end{array}\right]$$

rotates the strength vectors by the azimuthal angle *δ*.

As seen from the matrix representation, the azimuthal angle *δ* of cylindrical polarization does not affect the intensity distribution in an arbitrary transverse plane.

In the initial plane, polarization is linear in each point. Adopting an approach from [22], we can derive the polarization singularities index (Poincaré–Hopf index) [18] of the field (4). It is equal to the topological charge of the following scalar complex field:

(11) $$E_{c}=E_{x}+iE_{y}=\frac{e^{i\delta }}{q\sqrt{W_{0}}}\exp\left(-\frac{x^{2}+y^{2}}{qw_{0}^{2}}\right)\cos\left(\frac{x+iy}{\alpha_{0}q}\right),$$

In [15], the topological charge of such scalar fields was shown to be infinite, and therefore the Poincaré–Hopf index of the vector field (4) is also infinite.

## 3. Intensity Nulls of Light Fields with an Infinite Number of Polarization Singularities

Here we obtain the intensity nulls of the field (4). Since the determinants of both matrices are nonzero, for the zero intensity at some point, the following conditions should be fulfilled:

(12) $$\left\{\begin{array}{l} \cos\left(x_{+}+iy_{+}\right)=0,\\ \cos\left(x_{-}-iy_{-}\right)=0. \end{array}\right.$$

Both real and imaginary parts should be zero, thus we obtain

(13) $$\left\{\begin{array}{l} \cos x_{+}\cosh y_{+}=0,\\ \sin x_{+}\sinh y_{+}=0,\\ \cos x_{-}\cosh y_{-}=0,\\ \sin x_{-}\sinh y_{-}=0. \end{array}\right.$$

The hyperbolic cosine cannot be zero. Thus, from the first and third equation in (13) we determine that cos *x*_+_ = cos *x*_−_ = 0. This means that sin *y*_+_ ≠ 0 and sin *x*_−_ ≠ 0 and, therefore,

(14) $$\left\{\begin{array}{l} \cos x_{+}=0,\\ y_{+}=0,\\ \cos x_{-}=0,\\ y_{-}=0. \end{array}\right.$$

Since *y*_+_ = *y*_−_ = 0, we determine that *y* cos *ψ* = *x* sin *ψ* = 0.

In the initial plane, *ψ* = 0, thus the intensity nulls reside in points with the coordinates

(15) $$\left\{\begin{array}{l} x=\alpha_{0}\left(\pi /2+\pi p\right),\\ y=0. \end{array}\right.$$

In the far field, *ψ* → π/2, and the intensity nulls reside on the vertical axis in points with the coordinates

(16) $$\left\{\begin{array}{l} y=\alpha_{0}\left|q\right|\left(\pi /2+\pi p\right),\\ x=0. \end{array}\right.$$

For finite distances *z*, cos *ψ* ≠ 0 and sin *ψ* ≠ 0. Therefore, conditions (14) cannot be fulfilled and the field (4) does not have the intensity nulls.

Now we consider vicinities of the intensity nulls in the far field, i.e., points with the coordinates

(17) $$\left\{\begin{array}{l} x=\rho \cos\theta ,\\ y=\alpha_{0}\left|q\right|\left(\pi /2+\pi p\right)+\rho \sin\theta , \end{array}\right.$$

with ρ << *α*_0_, *w*_0_. Since *ψ* → *π*/2 in the far field, then in these points the rotated coordinates (6) read as

(18) $$\left\{\begin{array}{l} x_{\pm }=\pm \left(\pi /2+\pi p\right)\pm \rho \sin\theta /\left(\alpha_{0}\left|q\right|\right),\\ y_{\pm }=\mp \rho \cos\theta /\left(\alpha_{0}\left|q\right|\right). \end{array}\right.$$

Therefore, $x_{\pm }\pm iy_{\pm }\approx \pm \left(\pi /2+\pi p\right)-i\rho e^{\pm i\theta }/\left(\alpha_{0}\left|q\right|\right)$, $\cos\left(x_{\pm }\pm iy_{\pm }\right)\approx \pm {\left(-1\right)}^{p}i\rho e^{\pm i\theta }/\left(\alpha_{0}\left|q\right|\right)$, and the field amplitude is equal to:

(19) $$\begin{array}{l} E\left(\rho \cos\theta ,\alpha_{0}\left|q\right|\left(\pi /2+\pi p\right)+\rho \sin\theta ,z>>z_{0}\right)\\ \;\approx {\left(-1\right)}^{p}\frac{1}{2q\sqrt{W_{0}}}\frac{i\rho }{\alpha_{0}\left|q\right|}\exp\left(-\frac{\alpha_{0}^{2}{\left|q\right|}^{2}}{qw_{0}^{2}}\right)\left[\exp\left(i\delta +i\theta \right)\left(\begin{array}{c} 1\\ -i \end{array}\right)-\exp\left(-i\delta -i\theta \right)\left(\begin{array}{c} 1\\ i \end{array}\right)\right]\\ \;={\left(-1\right)}^{p}\frac{1}{q\sqrt{W_{0}}}\frac{\rho }{\alpha_{0}\left|q\right|}\exp\left(-\frac{\alpha_{0}^{2}{\left|q\right|}^{2}}{qw_{0}^{2}}\right)\left(\begin{array}{c} \cos\left(\theta +\delta +\pi /2\right)\\ \sin\left(\theta +\delta +\pi /2\right) \end{array}\right). \end{array}$$

This means that the azimuthal angle *δ* in the far field increases by *π*/2, i.e., radial polarization is converted to azimuthal and vice versa.

## 4. Intensity and Spin Angular Momentum Density Distribution of Light Fields with an Infinite Number of Polarization Singularities

The intensity distribution of the field (4) is given by (Appendix A):

(20) $$\begin{array}{l} I\left(x,y,z\right)={\left|E_{x}\left(x,y,z\right)\right|}^{2}+{\left|E_{y}\left(x,y,z\right)\right|}^{2}=\\ \;=\frac{1}{4{\left|q\right|}^{2}W_{0}}\exp\left(-2\frac{x^{2}+y^{2}}{{\left|q\right|}^{2}w_{0}^{2}}\right)\left(\cos2x_{+}+\cos2x_{-}+\cosh2y_{+}+\cosh2y_{-}\right). \end{array}$$

In the same way, we can derive the distribution of the longitudinal component of the SAM density:

(21) $$S_{z}\left(x,y,z\right)=\frac{1}{4{\left|q\right|}^{2}W_{0}}\exp\left(-2\frac{x^{2}+y^{2}}{w_{0}^{2}{\left|q\right|}^{2}}\right)\left(\cos2x_{-}+\cosh2y_{-}-\cos2x_{+}-\cosh2y_{+}\right).$$

Hence, both the SAM density and intensity distributions are independent of the azimuthal angle *δ* of cylindrical polarization. Note that although the scalar field (2) has an infinite topological charge, and the vector field (4) has an infinite polarization singularity index, the density of the SAM (21) has a finite value at each point, since it has a Gaussian envelope (hyperbolic cosines in (21) depend linearly on the argument). The expressions (20) and (21) allow for obtaining the coordinates of C-points of the field (4). C-points are points where the tilt of the major axis of the polarization ellipse in the beam cross section is undefined [18]. That is, C-points are points with circular polarization. For instance, right circular polarization appears in points where *S_z_* = *I*. In these points, $\cos2x_{+}+\cosh2y_{+}=0$ and, therefore, cos 2*x*_+_ = –1 and cosh 2*y*_+_ = 1, i.e., *y*_+_ = 0 and *x*_+_ = (*π*/2)(2*p* + 1), with *p* being an integer number. Then, the coordinates of the C-points are

(22) $$\left(\begin{array}{c} x_{\mathrm{RCP}}\\ y_{\mathrm{RCP}} \end{array}\right)=\frac{\pi }{2}\alpha_{0}\left|q\right|\left(1+2p\right)\left(\begin{array}{c} \cos\psi \\ \sin\psi \end{array}\right).$$

Similarly, left circular polarization appears in points with *S_z_* = −*I*. Coordinates of these points are equal to

(23) $$\left(\begin{array}{c} x_{\mathrm{LCP}}\\ y_{\mathrm{LCP}} \end{array}\right)=\frac{\pi }{2}\alpha_{0}\left|q\right|\left(1+2p\right)\left(\begin{array}{c} \cos\psi \\ -\sin\psi \end{array}\right).$$

**Figure 2:**
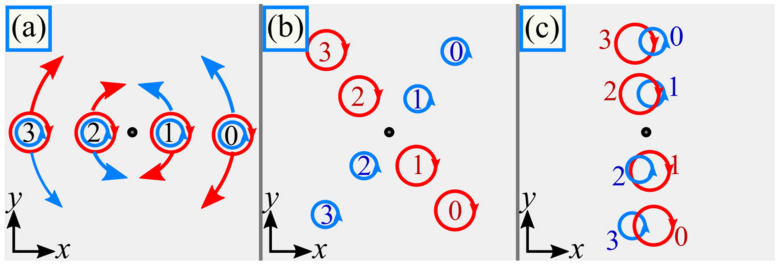
Mechanism of destruction of the polarization singularities after the initial plane and of their reconstruction in the far field. In the initial plane, points with left and right circular polarization coincide (**a**). Then, on propagation, they rotate around the optical axis in different directions (**b**), and in the far field they merge again (**c**).

The dynamics of the C-points explain the destruction of the polarization singularities after the initial plane and their reconstruction in the far field (Figure 2). Due to the splitting of left and right circular polarization, the beam (4) acquires nonzero SAM density upon propagation, and appearing areas with alternating SAM density manifest about the spin Hall effect.

We failed to derive exact expressions for the points of maximal SAM density. However, expressions (20) and (21) are simplified when *w*_0_ ≫ *α*_0_. Indeed, using identities for the sums and differences of trigonometric and hyperbolic functions, we obtain

(24) $$\begin{array}{l} I\left(x,y,z\right)=\frac{1}{2{\left|q\right|}^{2}W_{0}}\exp\left(-2\frac{x^{2}+y^{2}}{{\left|q\right|}^{2}w_{0}^{2}}\right)\\ \;\times \left[\cos\left(\frac{2x\cos\psi }{\alpha_{0}\left|q\right|}\right)\cos\left(\frac{2y\sin\psi }{\alpha_{0}\left|q\right|}\right)+\cosh\left(\frac{2x\sin\psi }{\alpha_{0}\left|q\right|}\right)\cosh\left(\frac{2y\cos\psi }{\alpha_{0}\left|q\right|}\right)\right], \end{array}$$

(25) $$\begin{array}{l} S_{z}\left(x,y,z\right)=\frac{1}{2{\left|q\right|}^{2}W_{0}}\exp\left(-2\frac{x^{2}+y^{2}}{w_{0}^{2}{\left|q\right|}^{2}}\right)\\ \;\times \left[\sin\left(\frac{2x\cos\psi }{\alpha_{0}\left|q\right|}\right)\sin\left(\frac{2y\sin\psi }{\alpha_{0}\left|q\right|}\right)+\sinh\left(\frac{2x\sin\psi }{\alpha_{0}\left|q\right|}\right)\sinh\left(\frac{2y\cos\psi }{\alpha_{0}\left|q\right|}\right)\right]. \end{array}$$

Products of two trigonometric or hyperbolic functions can be represented as a sum of four exponents. Thus, Equations (20) and (21) contain eight exponential terms. The first four terms do not exceed the value (8|*q*|^2^*W*_0_)^−1^, whereas the other four terms describe off-axis Gaussian beams:

(26) $$\begin{array}{l} GB_{\pm \pm }\left(x,y,z\right)=\frac{1}{8{\left|q\right|}^{2}W_{0}}\exp\left(-2\frac{x^{2}+y^{2}}{w_{0}^{2}{\left|q\right|}^{2}}\right)\exp\left(\pm \frac{2x\sin\psi }{\alpha_{0}\left|q\right|}\pm \frac{2y\cos\psi }{\alpha_{0}\left|q\right|}\right)=\\ =\frac{1}{8{\left|q\right|}^{2}W_{0}}\exp\left\{\frac{-2}{w_{0}^{2}{\left|q\right|}^{2}}\left[{\left(x-x_{c,\pm }\right)}^{2}+{\left(y-y_{c,\pm }\right)}^{2}\right]+\frac{w_{0}^{2}}{2\alpha_{0}^{2}}\right\}, \end{array}$$

with

(27) $$\begin{array}{l} x_{c,\pm }=\pm \frac{w_{0}^{2}\left|q\right|}{2\alpha_{0}}\sin\psi ,\\ y_{c,\pm }=\pm \frac{w_{0}^{2}\left|q\right|}{2\alpha_{0}}\cos\psi . \end{array}$$

These terms achieve values (8|*q*|^2^*W*_0_)^−1^exp[(*w*_0_/*α*_0_)^2^/2]. Thus, if *w*_0_ >> *α*_0_, the first four terms can be neglected. Then, the intensity and the SAM density (1) are equal to the intensity of the four Gaussian beams:

(28) $$I\left(x,y,z\right)=GB_{++}+GB_{--}+GB_{+-}+GB_{-+},$$

(29) $$S_{z}\left(x,y,z\right)=GB_{++}+GB_{--}-GB_{+-}-GB_{-+}.$$

If these four beams are far enough from each other (i.e., |*x*_c+_ − *x*_c−_| >> *w*_0_|*q*|, |*y*_c+_ − *y*_c−_| >> *w*_0_|*q*|), then these terms almost do not affect each other and, obviously, the points with the maximal SAM density coincide with the points of maximal intensity. In this case, the dependence of the maximal SAM density on the propagation distance *z* is given by

(30) $$\underset{x,y}{\max}\;S_{z}\left(x,y,z\right)\approx \frac{1}{8{\left|q\right|}^{2}W_{0}}\exp\left(\frac{w_{0}^{2}}{2\alpha_{0}^{2}}\right).$$

Hence, the SAM density decreases with the propagation distance from the initial plane in a similar law, as does the intensity in the Gaussian beam center. However, polarization is everywhere linear in the initial plane, i.e., the maximal SAM density is zero. This means that it at first increases near the initial plane and then, when the Gaussian beams are split, begins to decrease, i.e., the maximal SAM density is achieved in near-field diffraction.

## 5. Identification of Light Fields with an Infinite Number of Polarization Singularities

In our work [15], we studied an analogy between scalar fields with an infinite topological charge and conventional circularly symmetric optical vortices. Similarly, we can consider circular fields with cylindrical polarization and fields with an infinite number of polarization singularities. We suppose that a field with cylindrical polarization is composed of two circularly polarized single-ringed LG beams with opposite topological charges:

(31) $$\begin{array}{l} E\left(r,\varphi ,0\right)={\left(\frac{r}{w_{0}}\right)}^{m}\exp\left(-\frac{r^{2}}{w_{0}^{2}}+im\varphi \right)\left(\begin{array}{c} 1\\ -i \end{array}\right)+{\left(\frac{r}{w_{0}}\right)}^{m}\exp\left(-\frac{r^{2}}{w_{0}^{2}}-im\varphi \right)\left(\begin{array}{c} 1\\ i \end{array}\right)\\ \;=2{\left(\frac{r}{w_{0}}\right)}^{m}\exp\left(-\frac{r^{2}}{w_{0}^{2}}\right)\left[\begin{array}{c} \cos\left(m\varphi \right)\\ \sin\left(m\varphi \right) \end{array}\right]. \end{array}$$

where (*r*, *φ*) are the polar coordinates.

**Figure 3:**
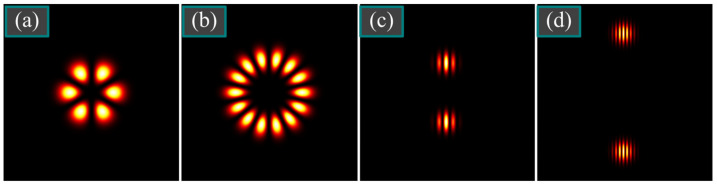
Intensity distribution of the *E_x_* component of two beams with cylindrical polarization (31) in the initial plane at *m* = 3 (**a**) and at *m* = 7 (**b**), as well of two beams with an infinite number of polarization singularities (4) at a different singularities density: *α*_0_ = *w*_0_/5 (**c**) and *α*_0_ = *w*_0_/10 (**d**).

Such a field can be easily identified using a polarizer. If it transmits only one polarization, then a multi-petal intensity distribution is obtained, which allows for determining the order of cylindrical polarization by counting the petals (Figure 3a,b).

Similarly, registering the intensity of only one transverse component of the field (4) allows for determining the density of polarization singularities. Indeed, in the initial plane, the field (4) can be written as

(32) $$\begin{array}{l} E_{x}\left(x,y,0\right)=\frac{1}{2\sqrt{W_{0}}}\exp\left(-\frac{w_{0}^{2}}{4\alpha_{0}^{2}}\right)\\ \;\times \left\{\exp\left[-\frac{x^{2}+{\left(y+w_{0}^{2}/\left(2\alpha_{0}\right)\right)}^{2}}{w_{0}^{2}}\right]+\exp\left[-\frac{x^{2}+{\left(y-w_{0}^{2}/\left(2\alpha_{0}\right)\right)}^{2}}{w_{0}^{2}}\right]\right\}\cos\left(\frac{x}{\alpha_{0}}\right). \end{array}$$

Hence, the intensity distribution of the *x*-component has vertical zero-intensity lines, whose frequency allows for determining the density of polarization singularities (Figure 3c,d).

## 6. Numerical Simulation

**Figure 4:**
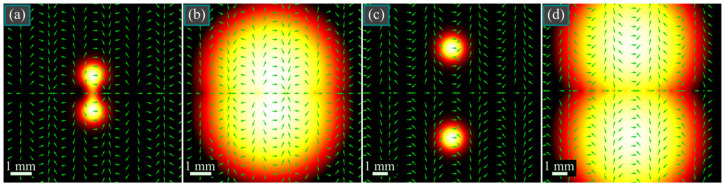
Intensity distributions of two beams (4) in the initial plane (**a**,**c**) and the logarithm of this distribution (**b**,**d**), as well as the polarization directions (green arrows) for the following parameters: wavelength λ = 532 nm, Gaussian beam waist radius *w*_0_ = 1 mm, and distance between the polarization singularities π*α*_0_ = π*w*_0_/2 ≈ 1.57 mm (**a**,**b**) and π*α*_0_ = π*w*_0_/5 ≈ 0.63 mm (**c**,**d**).

Shown in Figure 4 are intensity distributions of two beams (4) (with a different distance between the polarization singularities) in the initial plane, in uniform and in logarithmic color scale, as well as polarization directions. The intensity distributions were computed as *I*(*r*, *φ*, 0) = |*E_x_*(*r*, *φ*, 0)|^2^ + |*E_y_*(*r*, *φ*, 0)|^2^ using Equation (4), whereas the logarithmic distribution was computed as ln(10^−18^ + *I*(*r*, *φ*, 0)/max *I*(*r*, *φ*, 0)), where the constant 10^−18^ was introduced for avoiding the logarithm of zero in points with zero intensity. Polarization directions were computed by the formula arg(*E_x_*(*r*, *φ*, 0) + *iE_y_*(*r*, *φ*, 0)).

As seen in Figure 4, the intensity distribution has a shape of two light spots located symmetrically relative to the horizontal coordinate axis, and there are polarization singularities with radial polarization, residing periodically on this axis. Due to the low intensity, they are not visible, but they can be seen on the intensity distribution in logarithmic color scale.

**Figure 5:**
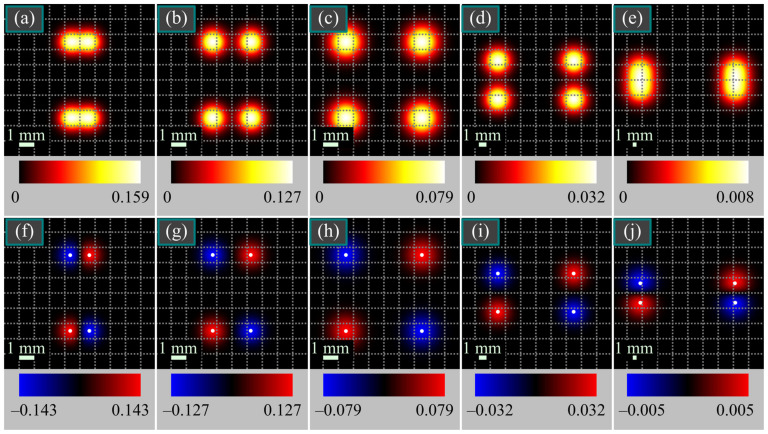
Intensity (**a**–**e**) and SAM density (**f**–**j**) distributions of the beam (4) from Figure 4c,d in several transverse planes for the following parameters: wavelength *λ* = 532 nm, Gaussian beam waist radius *w*_0_ = 1 mm, distance between the polarization singularities *πα*_0_ = *πw*_0_/5 ≈ 0.63 mm, and propagation distances from the initial plane *z* = *z*_0_/4 (**a**,**f**), *z* = *z*_0_/2 (**b**,**g**), *z* = *z*_0_ (**c**,**h**), *z* = 2*z*_0_ (**d**,**i**), and *z* = 5*z*_0_ (**e**,**j**). White dots on the SAM density distributions indicate the positions of maxima, obtained by Equation (27). The numbers near the color scales denote the minimal and maximal values.

Figure 5 illustrates the intensity and SAM density distributions of the vector beam from Figure 4c,d in several transverse planes.

According to Figure 5, both light spots split, each into two spots, one of which shifts to the left and the other shifts to the right. It is also seen that the maximal SAM density decreases upon propagation, which is consistent with Equation (30).

**Figure 6:**
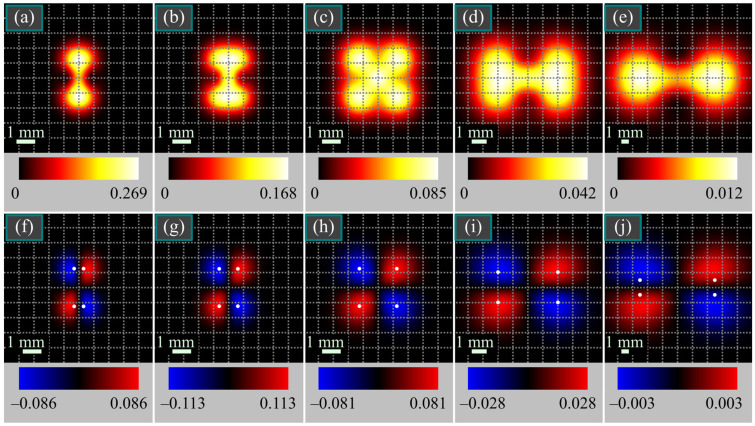
Intensity (**a**–**e**) and SAM density (**f**–**j**) distributions of the beam (4) from Figure 4a,b in several transverse planes for the following parameters: wavelength *λ* = 532 nm, Gaussian beam waist radius *w*_0_ = 1 mm, distance between the polarization singularities *πα*_0_ = *πw*_0_/2 ≈ 1.57 mm, and propagation distances from the initial plane *z* = *z*_0_/4 (**a**,**f**), *z* = *z*_0_/2 (**b**,**g**), *z* = *z*_0_ (**c**,**h**), *z* = 2*z*_0_ (**d**,**i**), and *z* = 5*z*_0_ (**e**,**j**). White dots in the SAM density distributions denote the positions of maxima computed by Equation (27). The numbers near the color scales denote the minimal and maximal values.

**Figure 7:**
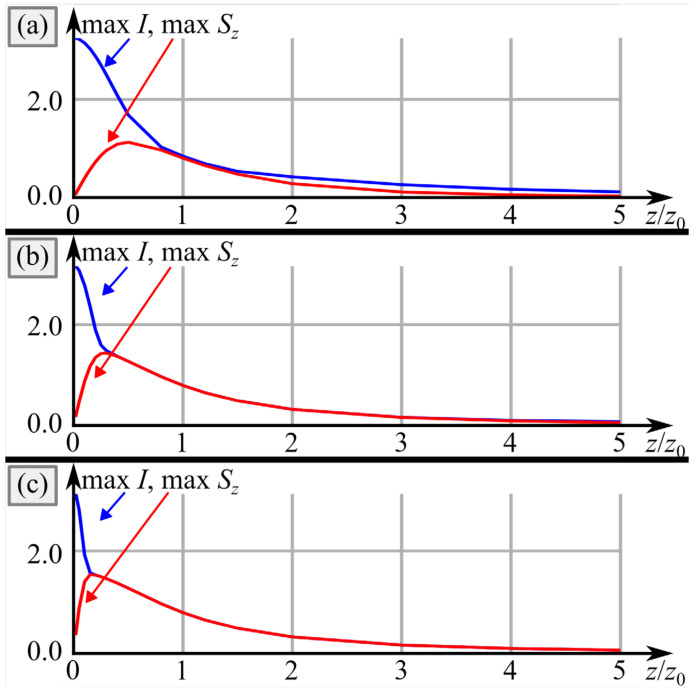
Dependence of the maximal intensity and of the maximal SAM density on the propagation distance for the distance between the polarization singularities equal to *πα*_0_ = *πw*_0_/2 ≈ 1.57 mm (**a**), *πα*_0_ = *πw*_0_/5 ≈ 0.63 mm (**b**), and *πα*_0_ = *πw*_0_/10 ≈ 0.31 mm (**c**).

Figure 6 depicts the intensity and SAM density distributions of the vector beam from Figure 4a,b in several transverse planes. In contrast to Figure 4c,d, light spots in Figure 4a,b are closer to each other; thus, they do not split so fast upon propagation into spots with left and right circular polarization, compared to Figure 5. Therefore, maximal SAM density in Figure 5 decreases immediately from *z* = *z*_0_/4 till *z* = 5*z*_0_, whereas in Figure 6 it at first increases at distances up to *z* = *z*_0_/2 and then decreases. The dependence of the maximal SAM density on the propagation distance is illustrated in Figure 7.

Figure 7 reveals that the maximal SAM density is achieved closer to the initial plane, when the distance between the polarization singularities decreases. This effect has a physical explanation since, as seen from Equation (4), decreasing value *α*_0_ leads not only to moving the light spots away from each other, but also to increasing space frequency along the axis *y*. Therefore, with decreasing *α*_0_, each light spot splits into two spots with opposite circular polarization faster.

## 7. Discussion

Usually, the spin Hall effect is considered in inhomogeneous media or in the presence of media interfaces, including metasurfaces. In most cases, the spin effect is associated with spin–orbit interaction. In this paper, we consider the propagation of a paraxial vortex laser vector beam in free space. The mechanism of appearance of the spin Hall effect in the considered beam is as follows. Beam (4) in the initial plane is a superposition of two vortex beams with left- and right-handed circular polarization, in which the centers of an infinite number of phase singularities are located at the same points on the horizontal axis, and have topological charges of different signs +1 and −1. The polarization of such a beam is linear in the initial plane, but as soon as the beam begins to propagate, optical vortices with left- and right-handed polarizations are separated and begin to rotate in different directions (vortices with a charge of −1 rotate clockwise, while those with a charge of +1 rotate counterclockwise). Therefore, regions with different spins are formed in the beam cross section, which leads to the Hall effect. The presence of an infinite number of screw dislocations in the initial beam is not fundamental; the number of vortices can be finite. Below, for comparison, we present an analysis of some works in which paraxial vortex beams were also considered, but with a finite number of polarization singularity points [14], with a fractional topological charge [23], and with vortex beams in a crystal [24].

In our recent work [14], we considered a paraxial laser beam with a finite number of polarization singularity points which are uniformly distributed over the beam cross section on a circle of some radius and in which the polarization is not defined. At all other points in the beam cross section in the initial plane, the polarization is linear. When such a beam propagates, each point of the polarization singularity in the beam cross section splits into two regions with elliptical polarization of a different sign, which move around the circle in different directions until they unite again at some distance and form points of the polarization singularity. The polarization becomes linear around these points. In this paper, we considered another paraxial beam, which is a superposition of optical vortices with left- and right-handed circular polarization and with an infinite number of phase singularities of different signs. In the initial plane, the centers of phase singularities for both beams coincide and are situated on the horizontal axis. In this case, the beam has a linear polarization at each point. When such a beam propagates in free space, then since the beams in superposition have topological charges of different signs, the centers of the phase singularities diverge and begin to rotate around the optical axis. In this case, the polarization in such parted centers became circular of different signs (C-points). In far-field diffraction, C-points of different signs coincide and form V-points, in which the polarization is undefined. Therefore, our previous work [14] is similar to this work in terms of the research method, but the considered beams are completely different.

In [23], a vector paraxial beam with an initial linear polarization and an initial fractional topological charge was also considered. The authors called this beam the erf-Gaussian beam. When it propagates in free space, a lot of optical vortices of different signs appear in the beam cross section, and the polarization near the intensity boundaries is elliptical of different signs. In fact, in [23], the authors showed that there is a spatial separation of the left- and right-handed circular polarizations in asymmetric paraxial beams with an initial linear polarization, which leads to the spin Hall effect.

In [24], a paraxial vortex laser beam propagating with a tilt to the axis of a birefringent crystal was considered. It was shown that two beams (ordinary and extraordinary) are formed in the crystal, each of which is a superposition of two optical vortices with topological charges +1 and −1 and with circular polarizations of different signs. It was also shown that in the cross section of such a beam there are points with circular polarization (C-points) of different signs. However, in these works [23,24] the SAM and OAM of the considered beams were not calculated.

## 8. Conclusions

In this work, we have constructed a vector Gaussian beam with an infinite number of polarization singularities residing on a straight line. For such a beam, the intensity distribution was derived analytically, and it turned out that the polarization singularities appear only in the initial plane and in the far field. We found that the polarization singularities index (Poincaré–Hopf index) is infinite. After propagation from the initial plane to the far field, the azimuthal angle of polarization singularities increases by *π*/2, i.e., initial radial polarization is converted into azimuthal and vice versa. We obtained a distribution of the longitudinal component of the spin angular momentum density. Similar to the intensity distribution, it is independent of the azimuthal angle of polarization singularities. When such a vectorial field propagates in free space, an infinite number of C-points appears, where polarization is circular. The distance to the transverse plane with the maximal spin angular momentum density decreases with as the distance between the polarization singularities decreases in the initial plane. Generation of alternating areas with left and right circular polarization, despite linear polarization in the initial plane, manifests in the optical spin Hall effect. Application areas of the results obtained include designing micromachines for optically driving microscopic objects. The SAM causes particles to rotate around their centers of mass and engineering the SAM density distribution of the studied light field can allow simultaneous manipulation of an ensemble of four particles. Another application is optical information transmission, where the density of polarization singularities can be used for encoding the data.

## Data Availability

Not applicable.

## Appendix A. Derivation of the Intensity Distribution

Since the azimuthal angle *δ* of cylindrical polarization does not affect the intensity distribution, we suppose for simplicity that *δ* = 0. Then, the transverse field components are given by

(A1) $$\left\{\begin{array}{l} E_{x}\left(x,y,z\right)=\frac{1}{2q\sqrt{W_{0}}}\exp\left(-\frac{x^{2}+y^{2}}{qw_{0}^{2}}\right)\left[\cos\left(x_{+}+iy_{+}\right)+\cos\left(x_{-}-iy_{-}\right)\right],\\ E_{y}\left(x,y,z\right)=\frac{-i}{2q\sqrt{W_{0}}}\exp\left(-\frac{x^{2}+y^{2}}{qw_{0}^{2}}\right)\left[\cos\left(x_{+}+iy_{+}\right)-\cos\left(x_{-}-iy_{-}\right)\right], \end{array}\right.$$

and the intensity distribution is

(A2) $$\begin{array}{l} I\left(x,y,z\right)={\left|E_{x}\left(x,y,z\right)\right|}^{2}+{\left|E_{y}\left(x,y,z\right)\right|}^{2}=\\ \;=\frac{1}{2W_{0}{\left|q\right|}^{2}}\exp\left(-2\frac{x^{2}+y^{2}}{{\left|q\right|}^{2}w_{0}^{2}}\right)\left[{\left|\cos\left(x_{+}+iy_{+}\right)\right|}^{2}+{\left|\cos\left(x_{-}-iy_{-}\right)\right|}^{2}\right]. \end{array}$$

Using an identity $\cos\left(x+iy\right)=\cos x\cosh y-i\sin x\sinh y$, we obtain

(A3) $$\begin{array}{l} I\left(x,y,z\right)=\frac{1}{2W_{0}{\left|q\right|}^{2}}\exp\left(-2\frac{x^{2}+y^{2}}{{\left|q\right|}^{2}w_{0}^{2}}\right)\\ \;\times \left[\cos^{2}x_{+}\cosh^{2}y_{+}+\sin^{2}x_{+}\sinh^{2}y_{+}+\cos^{2}x_{-}\cosh^{2}y_{-}+\sin^{2}x_{-}\sinh^{2}y_{-}\right]. \end{array}$$

Finally, using the formulae for trigonometric and hyperbolic functions with a double argument, we obtain an expression for the intensity distribution (20):

(A4) $$I\left(x,y,z\right)=\frac{1}{4W_{0}{\left|q\right|}^{2}}\exp\left(-2\frac{x^{2}+y^{2}}{{\left|q\right|}^{2}w_{0}^{2}}\right)\left[\cos2x_{+}+\cos2x_{-}+\cosh2y_{+}+\cosh2y_{-}\right].$$
